# *Monosolenium* (Marchantiopsida) Penetrates the Paleotropics

**DOI:** 10.3390/plants14121755

**Published:** 2025-06-08

**Authors:** Vadim A. Bakalin, Ksenia G. Klimova, Van Sinh Nguyen, Seung Se Choi

**Affiliations:** 1 Laboratory of Cryptogamic Biota, Botanical Garden-Institute FEB RAS, Vladivostok 690024, Russia; ksenia.g.klimova@mail.ru; 2 Institute of Biology, Graduate University of Science and Technology, Vietnam Academy of Science and Technology, Ha Noi 10072, Vietnam; nvsinh@vast.gov.vn; 3 Team of National Ecosystem Survey, National Institute of Ecology, Seocheon 33657, Republic of Korea

**Keywords:** Monosoleniaceae, East Asia, Indochina, Southeast Asia, distribution, rare species, complex thalloid liverworts, Hepaticae

## Abstract

East Asian *Monosolenium tenerum* is the only representative monotypic Monosoleniaceae and has been found for the first time in North Indochina in five provinces of Vietnam. All paleotropical localities, including those previously known in Indonesia, are situated not in the high mountains, as one might expect for the East Asian species occurring southward in East Asia, but in the lower altitude zones (i.e., in conditions physiognomically similar to where the species grows further north). A comparison of the bioclimatic parameters of locations where the species was found revealed clear similarities across all the Vietnamese localities of the species. The closest bioclimatic location to where the species has been found in Vietnam is in Nepal. The species reaches its greatest abundance in anthropogenically nitrified habitats, which may suggest that it is currently expanding to southern Indochina along the rural settlements in the catchments of the largest rivers and suggests that it may be discovered in Cambodia, Laos, and southern Vietnam in the near future.

## 1. Introduction

Many species that are typically considered Sino-Himalayan or East Asian in the broad sense have also been found in northern Vietnam along the Hoàng Liên Sơn Range and its spurs [1]. One of the taxa that we searched for in northern Vietnam was *Monosolenium tenerum* Griff., a unique complex thallose liverwort with a highly simplified structure. We ultimately found this species, but not where we expected. This account addresses the description of the distribution of this species in the Indochina, where this species was first found.

*Monosolenium tenerum* was described from the mountainous Assam as both a new species and a new genus in a paper published a few years after the author’s death [2]. Currently, this taxon is the only representative of the monotypic family Monosoleniaceae, which belongs to the order Marchantiales [3,4]. A detailed history of perturbations in the *Monosolenium tenerum* taxonomic position is provided by Pradhan et al. [5]. Bischler [6] published a distribution map of the genus based on the data relevant at that time ([6]: p. 54, map 14). Generally, this map is still appropriate today. An additional distribution map of the taxon is published online without any authority on the online resource [7]. The range of this species covers the northern part of India, Nepal, southern and southeastern China, Taiwan, and Japan, reaching central Honshu (note that source [7] provides all Japan as shaded, which is incorrect; the species starts to occur southward of the central Honshu). All the locations belong to the East Asian Floristic Region [8], otherwise known as the Eastern Asiatic Floristic Kingdom [9]. The new locations in Vietnam belong to a fundamentally different floristic kingdom—Paleotropis as defined in [8]. Miller et al. [10] have indicated *Monosolenium tenerum* also from Burma (=Myanmar), Thailand, Africa, Tonkin (=Vietnam), Java, and Hawaii. However, we did not find the relevant sources for those indications in the literature. In the sources cited above [5,6,7], this species is also not confirmed for listed areas. The recent checklist of Java Hepaticae [11] regards the report of *Monosolenium* for Java as the mistake. Staples and Imada [12] excluded it from Hawaii for a similar reason. In the present paper, we regard the data provided in Miller et al. [10] as the misprint. However, taking into account the new data here presented, the distribution of the taxon seems to be possible in those regions, with the possible exception of Africa. Moreover, Jayadi et al. [13] has indicated *Monosolenium* again for the anthropogenically transformed habitat in Indonesia. The latter paper was published in the journal with generally non-biodiversity scopes and the reports could not be clarified in the present study. The latter report is the only indicating *Monosolenium* for Paleotropics.

## 2. Results

The distribution of the species, according to the Table 1 and Table S1 (excluding locations in the greenhouses), extends from 36 to 20° N and from 81 to 140° E. Within this range, the species seems to avoid deep spreading into the mainland areas, apparently not occurring more than 500–700 km inland from the coasts of the Indian or Pacific Oceans. The species is quite thermophilic. The Annual Mean Temperature (BIO1) varies from 13 to 22 °C. In the northern parts of the species range (collection localities in Japan and Guizhou Province of China), the Temperature Seasonality (BIO4) is prominently expressed (700–800, unranked), but to the south (Taiwan and Vietnam), it drops to values of about 400. The Mean Temperature of Wettest Quarter (BIO8) varies from 20 to 26 °C, and the Driest Quarter (BIO9), which is often the coldest quarter in the species area, from 3.7 to 17 °C. The Annual Precipitation (BIO12) is quite high and varies from 1300 to 2500 mm per year, with maxima in southern Japan and Taiwan. At the same time, the amount of precipitation in the distribution area is strictly uneven and the difference between the Wettest Quarter (BIO16) and the Driest Quarter (BIO17) in most cases reaches several times: in the Driest Quarter it varies from 48 to 233 mm per quarter (only in the northern part of Taiwan it reaches 450 mm), while in the Wettest Quarter, it varies from 491 to 1304 mm per quarter. The Precipitation Seasonality (BIO15) varies from 28 in northern Taiwan to 94 (%) in Vietnam, which shows in the latter a great variability of precipitation between seasons. In West Nepal, the value of BIO15 exceeds 100% and is 128, which is rare and happens when the variance (standard deviation) of the precipitation throughout the year exceeds the average precipitation. Apparently, the reason is that the locality is far from coastal areas [14].

*Monosolenium tenerum* collection localities belong to only two climate types. All the Japanese specimens (1–7, from here on see Table 1) and most of the Chinese specimens (9, 12, 13) were collected in areas under a humid subtropical climate (Cfa) [15,16]; whereas all Vietnamese specimens (14–19), the West Nepal specimen (8), and two specimens from Taiwan (10, 11) are from areas with a monsoon subtropical climate (Cwa) [15,16]. Vietnamese specimens belong to the Paleotropis [8].

**Table 1:** List of specimens included in the analysis. The specimens were oriented from north to south (in order of decreasing latitude). The specimens collected by the authors are given in bold.

Specimen Number	Country	Region	References/Field IDs for Specimens Collected by Authors	Altitude, m a.s.l.	Latitude	Longitude
1	Japan	Ibaraki	[17]	30	36.200000	140.100000
2	Japan	Tokyo	[18]	29	35.700000	139.700000
3	Japan	Chiba	[19]	2	35.661752	139.892171
4	Japan	Chiba	[20]	40	35.369947	140.021753
5	Japan	Nara	[21]	10	34.584756	135.695123
6	Japan	Kochi	[22]	6	33.415672	133.302341
7	Japan	Miyazaki	[23]	100	31.626035	131.354674
8 ^1^	Nepal	West Nepal	[5]	120	28.462167	81.244667 ^1^
**13**	**China**	**Guizhou**	**China-53-20-13**	900	26.948000	106.981000
9	China	Taiwan	[24]	4	25.033289	121.517129
10	China	Taiwan	[25]	256	23.827847	120.802267
11	China	Taiwan	[26]	250	23.828667	120.801470
12	China	Taiwan	[27]	250	23.326528	121.228056
**14**	**Vietnam**	**Tuyên Quang**	**V-18-6-23**	339	22.342960	105.433930
**15**	**Vietnam**	**Tuyên Quang**	**V-19-1-23**	323	22.336050	105.431090
**16**	**Vietnam**	**Bắc Kạn**	**V-50-9-23**	283	22.32891	105.65849
**17**	**Vietnam**	**Phú Thọ**	**V-31-2-23**	449	21.112200	104.956090
**18**	**Vietnam**	**Thanh Hóa**	**V-29-5-22**	146	20.366770	105.449920
**19**	**Vietnam**	**Ninh Bình**	**V-24-1-22**	356	20.350290	105.593020

^1^ Apparently, the label of specimen examined in [5] has a typo in the degrees of longitude −87°, while the specimen was collected in the Royal Bardia National Park, which lies within only 81° of longitude [5]; therefore, we used the corrected coordinates.

**Figure 1:**
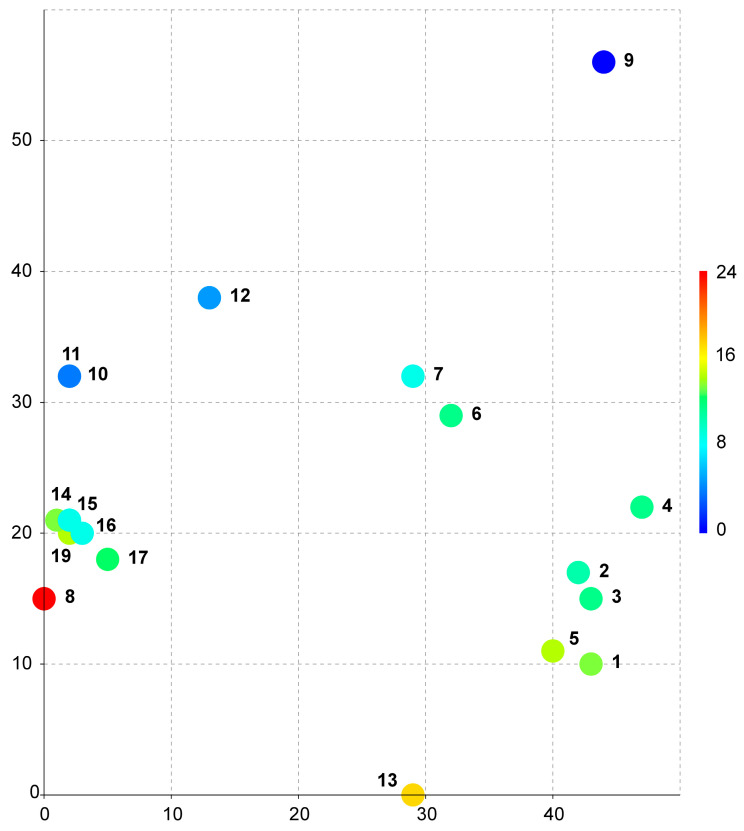
Scatter plot for the distribution of *Monosolenium tenerum* across bioclimatic parameters based on their collection localities across the area of the species according to Table S1. The numbers correspond to the number of *Monosolenium tenerum* specimens according to Table 1 (numbers from 14 to 19—specimens collected in Vietnam; the number 18 is coinciding with number 19 and cannot be shown in the scatter plot).

The data on the species distributions in Table 1 were used to identify the bioclimates listed in the accompanying Table S1. These data were used to construct a DCA bubble chart as described in the Section 4. The resulting diagram (Figure 1) illustrates the distribution of locations by climate. The coordinates of the three-dimensional grid are provided in Table 2.

**Figure 2:**
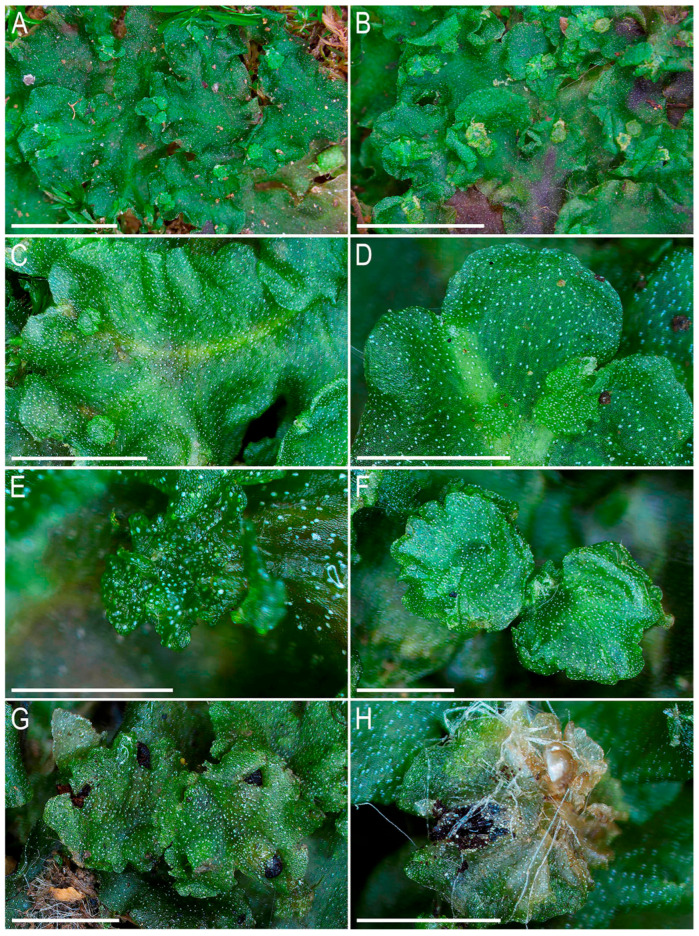
*Monosolenium tenerum* Griff.: (**A**,**B**) mat with female receptacles; (**C**) shoot with female receptacles; (**D**) shoot apex with female receptacles; (**E**,**F**) female receptacles; (**G**) mature sporangia in female receptacle; (**H**) female receptacle, spores from torn sporangium capsule. Scales: 10 mm for (**A**,**B**); 5 mm for (**C**); 3 mm for (**D**); 1 mm for (**E**–**H**). (**A**,**B**) from China-53-25-13 (VBGI), (**C**,**E**,**F**,**H**) from V-50-10-23 (VBGI, NH), (**D**) from V-18-7-23 (VBGI, NH), (**G**) from V-31-2-23 (VBGI, NH).

**Figure 3:**
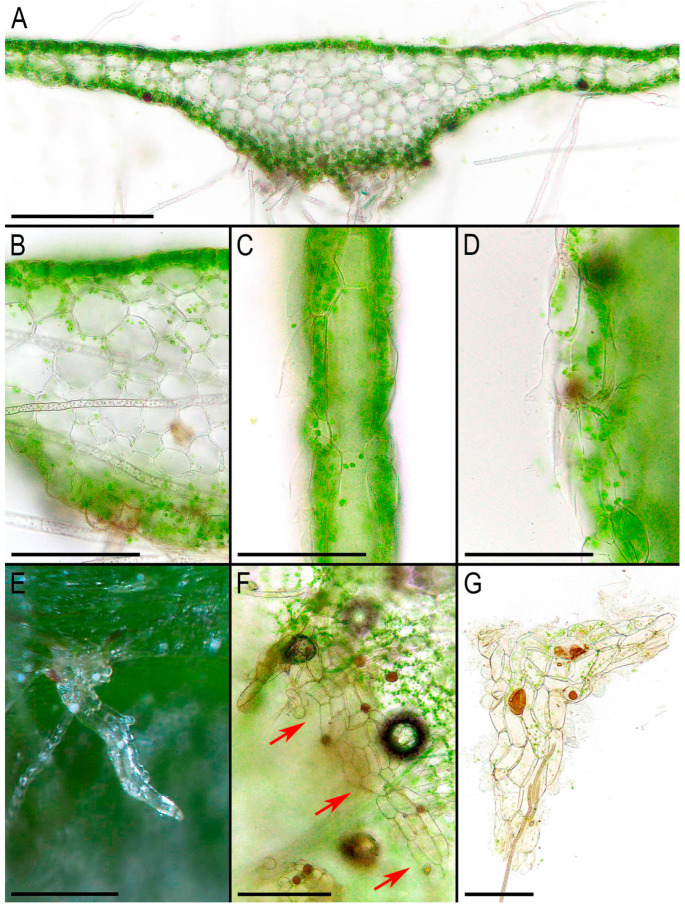
*Monosolenium tenerum* Griff.: (**A**) cross-section of the thallus; (**B**) cells in the middle part of the cross-section of the thallus; (**C**) cells in the ‘wing’ part of the cross-section of the thallus; (**D**) one layer of cells in thallus margin; (**E**) ventral scale; (**F**,**G**) cells of ventral scale. Scales: 500 µm for (**E**); 300 µm for (**A**,**F**); 100 µm for (**B**–**D**,**G**). All from V-50-9-23 (VBGI, NH).

**Figure 4:**
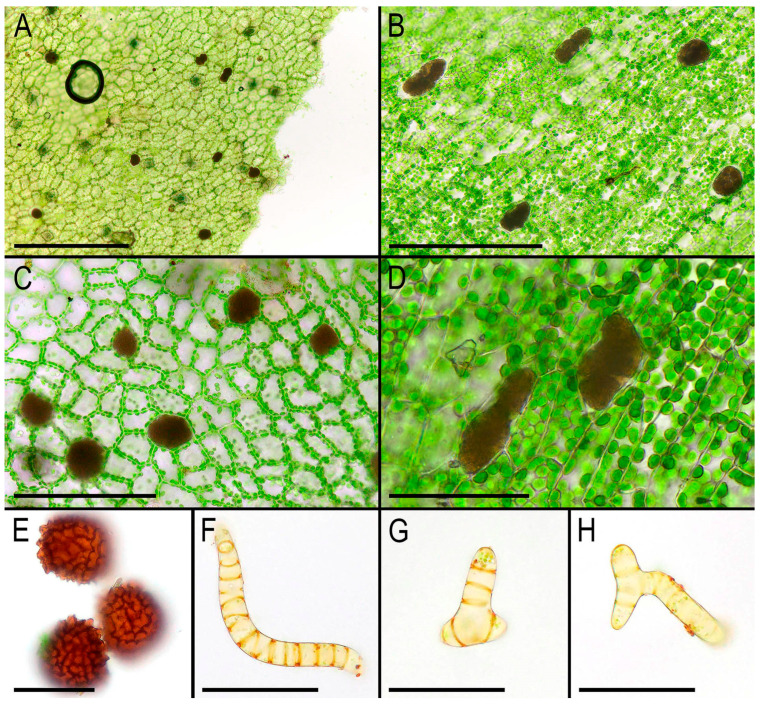
*Monosolenium tenerum* Griff.: (**A**–**D**) cells of the thallus; (**E**) mature spores; (**F**–**H**) elaters. Scales: 300 µm for (**A**); 100 µm for (**B**,**C**); 50 µm for (**E**,**F**); 30 µm for (**D**). (**A**) from China-53-25-13 (VBGI), (**B**,**D**) from V-24-20-22 (VBGI, NH), (**C**) from V-18-7-23 (VBGI, NH), (**E**–**H**) from V-19-2-23 (VBGI, NH).

The first description by Griffith [2] was unusually detailed for that time, and subsequently the morphology of *Monosolenium tenerum* has been repeatedly described and photographed. In the last three decades, detailed descriptions, line-art figures, and photographs have been provided by Bischler [6], Pradah et al. [5], and Singh & Singh [28]. An earlier detailed description was provided by Inoue [29], who first described Monosoleniaceae as a new family. Crandall-Stotler et al. [30] (p. 174) provided a review of the liverwort systematic supply describing the characteristic features of Monosoleniaceae: “Thallus undifferentiated, without air pores; ventral scales in 2 rows, with or without 1 small appendage; perigonal chambers aggregated in dorsal cushions on the thallus; sporophytes on stalked receptacles, with the receptacles lacking air pores and the stalk with 2 rhizoid furrows; involucres bivalved; pseudoperianths absent; seta remaining short; capsule dehiscence by irregular valves; specialized asexual structures absent”. Thus, there is no reason to provide another description here. However, we expect that providing photographs of the taxon obtained from living plants in Figure 2, Figure 3 and Figure 4 is practical. Many photographs that can be found in the literature are made from dead material and differ from what is observed in natural conditions with the hand lens. On the other hand, some photos available in GBIF were made in nature, but the purpose of the photographing is to represent the habitat, but not the morphology of the plants, which is impossible to discern in “natural” photographs. In addition, the basis for providing microphotographs is the still poor study of the variability of the structures of this species, especially its generative sphere. When searching in the field, this species is immediately clearly identified under a hand magnifying lens due to the white (brown in the transmissive light in the microscope slide) oil bodies filling the lumens of specialized cells (one large oil body per cell). Similar ‘spotting’, as far as we remember, is also characteristic of representatives of Treubiaceae, a morphologically very distant family.

## 3. Discussion

Despite the simple structure of the thallus, *Monosolenium* does not occupy a basal position in the Marchantiales phylogenetic system according to the available reconstructions. For example, according to [31], it is found in a sister branch to the Corsiniaceae + Exormothecaceae + Cyathodiaceae macroclade. According to the chronogram [31] (p. 1740, Figure 3), Monosoleniaceae evolved from other related groups at approximately 150 MA at the end of the Jurassic Period. Similar to that of [31], a phylogenetic topology was obtained by Xiang et al. [32] (p. 653), where the family is placed in Marchantiales, to the first of five major clades (clades A–E; Figure 2 in [32]), composed of six families: Cleveaceae, Corsiniaceae, Cyathodiaceae, Monosoleniaceae, Targioniaceae, and Wiesnerellaceae.

*Monosolenium* is a conditionally ‘weedy’ species that prefers nitrogen-rich habitats that are often of anthropogenic origin; this is described in detail by Schuster [33] (p. 20): “In Japan the incidence of this species has declined in the countryside in recent decades-after adoption of modem plumbing. When the old-fashioned privy was current, *Monoselenium* was a common “weed,” e.g., around the privies in the periphery of the Mossy Temple at Kyoto and in settled areas”. However, there are no such observations in the papers by Pradah et al. [5] for Nepal and Singh & Singh [28] for India. In these locations, the species was found either on the banks of a stream or along roads. Meantime, our observations coincide with those of Schuster [33]. Our collection in Guizhou Province cited in Table 1 was made off the trail in an area often used by tourists as an improvised toilet and is characterized by a strong smell of ammonia. The Vietnamese Ninh Bình Province collection was also made near a small tourist lodge on clayey soil with a strong smell of ammonia. This location was probably also used by tourists as a place for spontaneous toileting. The Tuyên Quang Province and Phú Thọ Province specimens were gathered on clay trail-sides where high concentrations of ammonia are not obvious. Moreover, the Thanh Hóa Province site was located on exposed rock near a waterfall: a place that is not likely used as a spontaneous toilet. The same can be said for Bắc Kạn Province (wet rocks near a stream). Thus, our findings agree with Schuster [33], that the species is rare in wild environments but becomes abundant where there is obvious (anthropogenic or not) nitrification. Another reason for the wide distribution of *Monosolenium* (and current range expansion) may be the apparent ease with which the taxon forms sporophytes and produces spores. This, coupled with its tendency to be common in anthropogenically nitrified habitats, may be mutually reinforcing factors. The role of both factors could not be statistically reliably assessed within the present study.

*Monosolenium tenerum* is a species of mainly lower and less commonly middle altitude levels in mountains. In India [28], the altitudinal range of the species extends from 550 to 1000 m a.s.l. In Nepal [5], the species was found to be very low for a mountainous country—only 120 m a.s.l. Specimens from the GBIF were collected at altitudes less than 400 m a.s.l. [17,18,19,20,21,22,23,24,25,26,27]. One of the “highest” localities of the species is our collection in Guizhou Province, China, where the species was found at 900 m a.s.l. This locality was previously discussed by Bakalin et al. [34]. The Vietnamese localities, although they are geographically southernmost in the general range of the species, are also found at lower altitude levels, within the range of 146 to 449 m a.s.l. (see specimens examined list in the Section 4). Owing to the nature of the vegetation, most of the natural habitats lie in the subtropics of East Asia. However, in the northern localities in China, and in Japan, the species is found in warm-temperate vegetation communities. At the same time, in northern Vietnam, where the species is found, typical tropical forests have already developed. Throughout the observed localities, the climate possesses seasonal features (at least dry/wet, less cool/warm).

As seen from the provided bubble chart (Figure 1), all collection sites in Vietnam are combined into one very dense cluster (points 14–19 in the diagram), despite the significant difference in geographic location, which exceeded 200 km. The only closest point to the Vietnamese cluster is the location in Nepal (point 8 in the diagram). The other nearest locations are points 10 and 11 from Taiwan. Based on the data presented in Supplementary Table S1, these locations (including those from Nepal) are all very similar in terms of all the parameters, including the average annual temperature, seasonality of precipitation and temperature, and absolute values for the wettest/driest and coldest/warmest quarters and belong to one type of climate—monsoon subtropical climate (Cwa) [15,16].

As we noted at the beginning of this paper (the last paragraph of the introduction), according to the data available before our studies, the species had a clearly expressed East Asian distribution which approached the borders of Indochina from the north (thus Southeast Asian floristic region [8]) but not did not penetrate there. Considering how many species penetrate the Tropic of Cancer along the Hoàng Liên Sơn Range from East Asia to Southeast Asia (both liverworts: [1], and vascular plants: [35]), we assumed that *Monosolenium* would be found there, at least in limited areas. However, our expectations were completely unfounded. We never found this species in the northern provinces of Vietnam, such as Lào Cai and Lai Châu, which are especially rich in Sino-Himalayan species. The expectation that this species would be located in the mountains was generally erroneous as the species does not seem to be associated with the mountainous landscapes (and therefore is not Sino-Himalayan in general). In contrast, the species is found at lower altitudes and continues to be found at lower elevations even south of the Tropic of Cancer. This circumstance makes it difficult to predict the potential distribution of the taxon in adjacent regions. If the species is at least partially associated with anthropogenically nitrified habitats, then we can expect its wide expansion in the rural settlements along the lower altitude levels of the Kong River catchment (which we have observed) and the Mekong River basin in general. In this case, its discovery in Laos, Cambodia, and southern part of Vietnam is expected in the near future. It should be noted that, since the species spreads southward, although not always, in many cases through human-transformed habitats, its distribution may not be completely subordinate to the peculiarities of the climate. In this case, not entirely suitable climatic conditions can be compensated for by significant nitrification of habitats, which allows the species to grow in places having a climate where it could not otherwise be found.

## 4. Materials and Methods

**Figure 5:**
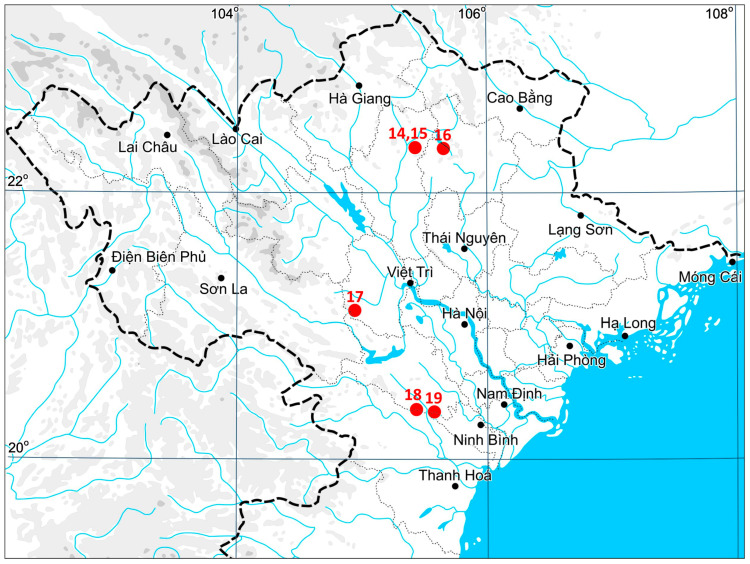
Dot distribution map of *Monosolenium tenerum* in Vietnam based on collected specimens. The numbers of localities correspond to Table 1 and Table S1.

The materials used in the study were collected from 2022 to 2023 in five provinces of northern Vietnam: Ninh Bình, Thanh Hóa (both from the Red River Delta Region in North Vietnam), Tuyên Quang, Phú Thọ, and Bắc Kạn (Northeast Vietnam). A distribution dot map of the species in Vietnam is provided in Figure 5. During collection, various parameters, including geographical coordinates, altitude, community type, substrate, and lighting conditions, were noted. All specimens were assigned unique field numbers. The specimens collected were studied at the Department of Ecology and Remote Sensing at the Institute of Biology of the Vietnam Academy of Science and Technologies (Hanoi, herbarium acronym HN), where intravital *Monosolenium* photographs were taken via digital cameras mounted on Nikon SMZ800N (Nikon Corporation, Shanghai, China) and Olympus BX43 (Olympus corporation, Tokyo, Japan) microscopes.

In an attempt to clarify the climatic conditions of the taxon, we used GBIF facilities since they cite many specimens of the genus [17,18,19,20,21,22,23,24,25,26,27], for which coordinates are measured. Therefore, we selected all unique precisely measured coordinates (excluding data originating from the greenhouses), and after combining them with our own data, subsequently identified 19 bioclimates from WorldClim [36,37] for them (the list of all data on the precise locations of the species involved in the analysis is provided in Table 1). The obtained bioclimatic data (Table S1) were analyzed via detrended correspondence analysis (DCA) via Past ver. 4.03c [38]. The DCA results were visualized in a three-dimensional grid graph, with the third dimension represented by a color gradient. The climate types were identified according to a climate map [15,16] based on Köppen–Geiger climate classification.

The species was found in five provinces that belong to two macro-regions of northern Vietnam (Northeast Vietnam, Red River Delta Region). Additionally, we included a specimen from the Guizhou Province of China that was collected in the course of our exploration in 2013 and discussed previously [34]. The list of the specimens examined is provided below.

Specimens examined:

VIETNAM, Northeast Vietnam, Tuyên Quang Province, Na Hang District, Na Chang Village, Na Hang Nature Reserve (22°20′34.66″ N 105°26′02.14″ E), 339 m a.s.l., evergreen tropical forest in narrow valley surrounded by limestone cliffs, partly shaded moist clayish trail side, 28 March 2023, V.A. Bakalin & M.H. Nguyễn V-18-6-23, V-18-7-23 (VBGI, HN); *ibid*. (22°20′09.78″ N 105°25′51.94″ E), 323 m a.s.l., partly shaded moist clayish soil near the stream 28 March 2023, V.A. Bakalin & M.H. Nguyễn V-19-1-23, V-19-2-23 (VBGI, HN); *ibid*. Bắc Kạn Province, Ba Bể District, Ba Bể National Park (22°19′44.07″ N 105°39′30.57″ E), 283 m a.s.l., wide stream valley with a waterfall and secondary bamboo thickets around, open wet cliff near the stream, 25 April 2023, V.A. Bakalin & M.H. Nguyễn V-50-9-23, V-50-10-23 (VBGI, HN); *ibid*. Phú Thọ Province, Tân Sơn District, Xuan Son National Park (21°06′43.92″ N 104°57′21.93″ E), 449 m a.s.l., evergreen tropical forest in a valley with many limestone outcrops, partly shaded moist trail cut, 4 April 2023, V.A. Bakalin & M.H. Nguyễn V-31-2-23, V-31-3-23 (VBGI, HN); Red River Delta Region, Thanh Hóa Province, Thạch Thành District, Mây waterfall surroundings (20°22′00.36″ N 105°26′59.71″ E), 146 m a.s.l., waterfall stairs over dense clay covering limestone outcrops, open wet cliffs near stream, 19 May 2022, V.A. Bakalin & M.H. Nguyễn V-29-5-22 (VBGI, HN); *ibid*. Ninh Bình Province, Nho Quan District, Cúc Phương National Park (20°21′01.03″ N 105°35′34.88″ E), 356 m a.s.l., tropical forest with limestone outcrops, clayish soil near small house in forest (probably with a lot of nitrogen), 16 May 2022, V.A. Bakalin & M.H. Nguyễn V-24-1-22, V-24-20-22 (VBGI, HN).

CHINA, Guizhou Province, Kaijang County, Nanjiang Gorge (26°56′52.5″ N 106°58′51.7″ E), 900 m a.s.l., broadleaved (mostly evergreen) forest on steep slope and within valley of stream, moist clay on steep slope, 20 November 2013, V.A. Bakalin China-53-20-13, China-53-22-13, China-53-23-13, China-53-25-13 (VBGI).

## 5. Conclusions

*Monosolenium tenerum* was found locally abundant in North Vietnam, which is the second country after the first Paleotropic report in Indonesia. The report, considering the pattern of its previously known locations, was not likely unexpected. Moreover, the bioclimatic characteristics of the collection sites in Vietnam relative to the bioclimatic parameters in other areas where the species has been found are somewhat different. The bioclimatically closest location to that found in Vietnam is the location of the species in Nepal. In contrast to expectations, the species was found not in the upper- or middle-altitude zones but at the lowest levels in the tropical forest zone. Given these locations, a wider distribution of the species in Indochina in general may be expected; this may also be facilitated by the nature of the species, which tends to occupy highly nitrified (including anthropogenic in origin) habitats. The environments of several known localities in Vietnam and China feature artificial (anthropogenic) nitrification. *Monosolenium tenerum* is not a Sino-Himalayan montane species but belongs to a group of subtropical thermophilic East Asian taxa at lower elevations. A number of these are already known south of the Tropic of Cancer, and many more may be expected there in further studies.

In addition to various anthropogenic impacts (development of the transport network, accessibility of remote settlements, lifestyle changes), the distribution of *Monosolenium* may theoretically be seriously affected by local or global climate changes. This issue could not be satisfactorily analyzed in the course of the present research, but the influence of such changes may be minimal, since due to the increase in average temperature, it was fashionable to assume the movement of this genus distribution to the north, and not to the south, as evidenced here.

## Figures and Tables

**Table 2:** Normalized values of DCA (unranked) for each compared *Monosolenium tenerum* specimen. The numbers of specimens collected in Vietnam are given in bold.

Specimen Number	Axis 1	Axis 2	Axis 3
1	43	10	14
2	42	17	11
3	43	15	12
4	47	22	12
5	40	11	15
6	32	29	12
7	29	32	9
8	0	15	25
9	44	56	0
10	2	32	4
11	2	32	4
12	13	38	5
13	29	0	18
**14**	2	21	9
**15**	2	21	9
**16**	3	20	9
**17**	5	18	13
**18**	1	21	14
**19**	2	20	15

## Data Availability

The original contributions presented in this study are included in the article. Further inquiries can be directed to the corresponding author(s).

## Supplementary Materials

The following supporting information can be downloaded at https://www.mdpi.com/article/10.3390/plants14121755/s1, Table S1: Obtained bioclimatic data for collecting localities of *Monosolenium tenerum* Griff. involved into the analysis.

## Abbreviations

The following abbreviations are used in this manuscript:

FEB RAS	Far Eastern Branch of the Russian Academy of Sciences
VBGI	Herbarium of the Botanical Garden-Institute FEB RAS, Vladivostok
NH	Herbarium of the Institute of Biology of the Vietnam Academy of Science and Technology, Hanoi
